# No Association Between Cyclosporine Levels and Dyslipidemia?

**DOI:** 10.5812/numonthly.14296

**Published:** 2014-01-13

**Authors:** Guido Filler

**Affiliations:** 1 Department of Pathology and Laboratory Medicine, University of Western Ontario, Ontario, Canada; 2 Department of Pediatrics, University of Western Ontario, Ontario, Canada; 3 Department of Medicine, University of Western Ontario, Ontario, Canada

**Keywords:** Kidney Transplantation, Cholesterol, Triglycerides, Cyclosporine

***Dear Editor,***

The recent manuscript analyzing whether there was a correlation between cyclosporine levels and dyslipidemia after renal transplantation in 1391 kidney transplant recipients (1) was read with great interest. Dyslipidemia is a common problem after renal transplantation and a potentially modifiable factor (2). This is particularly important because cardiovascular morbidity significantly contributes to the below-average life expectancy found in renal transplant patients (3).

In their cross-sectional study of 1391 subjects, Hosseini et al. found a very high prevalence of hypercholesterolemia (58.8%) and hypertriglyceridemia (86.6%) (1). Using univariate correlation analysis, they found only a weak correlation between cyclosporine levels two hours post-intake (C2) and dyslipidemia (Pearson correlation coefficient 0.18 and 0.16 for hypercholesterolemia and hypertriglyceridemia, respectively). C2-monitoring is the preferred method for assessing cyclosporine exposure, although there are some challenges with timely C2 blood sampling, and some patients may suffer toxicity or rejection when switching from trough level (C0) to C2 monitoring (4). Using logistic multivariate regression, only serum creatinine was associated with hyperlipidemia. Hosseini’s study did not assess the correlation of steroid levels or the steroid dose and dyslipidemia, even though steroid therapy is a well-known risk factor for hyperlipidemia and steroid avoidance has been a powerful tool to reduce the prevalence of hyperlipidemia in liver transplantation (5).

Hosseini’s study has several other limitations. No validation of the fasting state occurred. The results of the logistic regression were not well documented, and it is unclear what factors were included in the multivariate analysis. The limitations of the cross-sectional retrospective study are not well discussed, and such a study can only assess an association, no causality. Nonetheless, the stronger correlation of the hypertriglyceridemia with the GFR (measured as serum creatinine) is interesting; it is disappointing that targeting optimal calcineurin inhibitor exposure has little effect on hyperlipidemia. The authors call for prospective trials targeting better lipid control, with the hope that long-term outcomes will improve.

The authors are correct in their assessment that chronic kidney disease (CKD)-related complications after renal transplantation are poorly managed (3, 6, 7). Often, there is undertreatment of dyslipidemia. Longevity after renal transplantation could be improved significantly if similar multidisciplinary clinics were introduced as for CKD in the primary kidneys (8). It is of the utmost importance to determine whether targeting conventional cardiovascular risk factors can effectively modify cardiovascular morbidity (9). In a large study of national data, Sciarretta et al. found no association of renal damage with cardiovascular disease and the individual cardiovascular risk profile (9). It is also important to determine which intervention is most effective and what novel therapies can be employed to lower triglycerides. There is no known effective treatment of hypertriglyceridemia. Dietary approaches such as supplementation with omega-3 fatty acids should be studied prospectively (10).

Nonetheless, the undersigned is delighted that the attention is shifting towards modifiable long-term complications after renal transplantation. Cardiovascular risk factors are among the most significant factors affecting long-term outcomes in renal transplant recipients and are responsible for deaths with functioning graft. CKD is a major risk factor for cardiovascular morbidity following transplantation, and has a high prevalence in both renal and non-renal transplant patients. The relationship between impaired GFR and dyslipidemia needs to be studied further and effective therapeutic interventions have to be found.
